# The Dual Molecular Identity of Vestibular Kinocilia: Bridging Structural and Functional Traits of Primary and Motile Cilia

**DOI:** 10.1101/2025.04.16.647417

**Published:** 2025-06-23

**Authors:** Zhenhang Xu, Amirrasoul Tavakoli, Samadhi Kulasooriya, Huizhan Liu, Shu Tu, Celia Bloom, Yi Li, Tirone D. Johnson, Jian Zuo, Litao Tao, Bechara Kachar, David Z. He

**Affiliations:** 1 Department of Biomedical Sciences, Creighton University, Omaha, Nebraska 68178; 2 Section of Cell Biology, National Institute on Deafness and Other Communication Disorders, National Institutes of Health, Bethesda, Maryland 20892

**Keywords:** Vestibule, cochlea, hair cell, kinocilia, motile cilia, axoneme, transcriptome, mouse, bullfrog, human

## Abstract

Vestibular hair cells (HCs) convert gravitational and head motion cues into neural signals through mechanotransduction, mediated by the hair bundle—a mechanically integrated organelle composed of stereocilia and a kinocilium. The kinocilium, a specialized form of primary cilium, remains incompletely defined in structure, molecular composition, and function. To elucidate its characteristics, we conducted single-cell RNA sequencing of adult vestibular and cochlear HCs, uncovering a selective enrichment of primary and motile cilia–associated genes in vestibular HCs, particularly those related to the axonemal repeat complex. This enrichment of orthologous axonemal-related genes was conserved in zebrafish and human vestibular HCs, indicating a shared molecular architecture. Immunostaining validated the expression of key motile cilia markers in vestibular kinocilia. Moreover, live imaging of bullfrog and mouse HCs from crista ampullaris revealed spontaneous kinociliary motion. Together, these findings define the kinocilium as a unique organelle with molecular features of primary and motile cilia and support its previously unknown role as an active, force-generating element within the hair bundle.

## INTRODUCTION

The mammalian inner ear contains auditory and vestibular end organs to detect sound and motion respectively. Hair cells (HCs), the receptor cells in the inner ear, transduce mechanical stimuli in the form of sound or head movement into electrical signals (Fettiplace, 2017; Gillespie and Müller, 2009). The auditory sensory epithelium in the cochlea contains two types of HCs, the inner and outer HCs (IHCs and OHCs)(Dallos, 1992). The vestibular end organs comprise two otolith organs (utricular and saccular maculae) and three cristae associated with semicircular canals. Similar to the cochlea, the vestibular sensory epithelium consists of two types of HCs known as type I and type II based on morphology, physiology, and innervation (Eatock et al., 1998; Eatock and Songer, 2011).

Although mechanotransduction is a shared feature of all HCs, mammalian cochlear and vestibular HCs differ in morphology and function. One key difference lies in the structure of the hair bundle, which harbors specialized machinery for mechanotransduction. The hair bundle of adult vestibular HCs is composed of actin-rich stereocilia connected via lateral links to a single microtubule-based kinocilium, meanwhile cochlear HCs lose their kinocilia during HC maturation (Leibovici et al., 2005). Kinocilia are highly conserved in non-mammalian vertebrates (Leibovici et al., 2005) and play a pivotal role in establishing hair bundle polarity and mediating Hedgehog and WNT signaling during HC development (Moon et al., 2020; Shi et al., 2022). Despite possessing features of motile cilia such as the canonical “9+2” microtubule arrangement, the kinocilium has long been regarded as a specialized primary cilium (Kikuchi et al., 1989; Wang and Zhou, 2021). While the kinocilium contributes to the bundle mechanics (Baird, 1994; Kindt et al., 2012; Spoon and Grant, 2011), the molecular basis and function of this unique organelle in adult vestibular HCs remain unknown.

In the current study we utilized single-cell RNA-sequencing (scRNA-seq) to examine the transcriptomes of 1,522 HCs isolated from cochlear and vestibular sensory epithelia of adult CBA/J mice. Comparisons of the molecular profiles identified novel marker genes as well as shared and unique genes associated with mechanotransduction, ion channels, and pre- and post-synaptic structures in the IHCs, OHCs, type I and type II HCs. Notably, our analysis revealed a significant enrichment of genes related to primary and motile cilia in vestibular HCs, particularly those linked to the 96-nm axonemal repeat complex, a hallmark feature of motile cilia. Orthologous axonemal-related genes were also detected in zebrafish HCs and human vestibular HCs. We utilized transmission electron microscopy (TEM) to examine the ultrastructure of kinocilia and immunostaining to detect expression of key motile cilia proteins in vestibular HCs. We also used live imaging to examine kinocilia motion in bullfrog and mouse crista ampullaris. We modeled the atomic architecture of the 96-nm repeat, the core framework of the kinocilium axoneme. Together, these findings establish the kinocilium as a distinct organelle with molecular hallmarks of motile cilia and suggest it functions as an active, force-generating hair bundle component, influencing the mechanosensitivity of the kinocilium-bearing HCs across nonmammalian and mammalian species. In addition, our transcriptomic analysis provides new insight into the molecular mechanisms underlying phenotypical differences among the four different HC types in the adult mouse inner ear.

## RESULTS

**Fig. 1:**
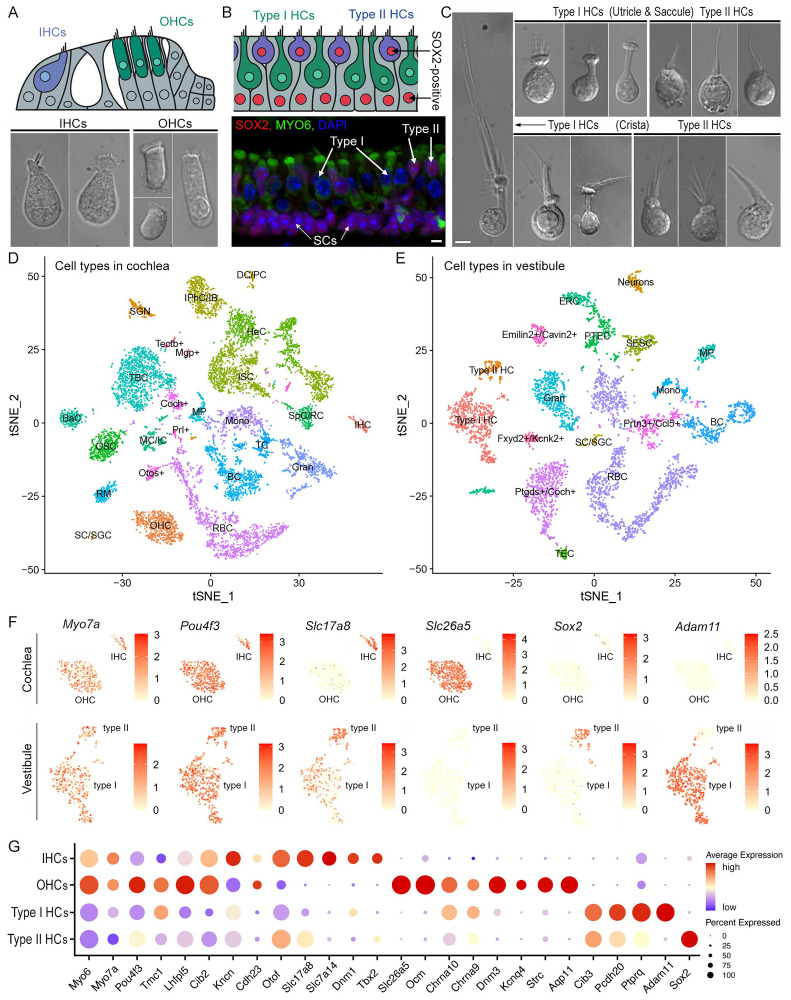
Single-cell transcriptional atlas of cochlear and vestibular cells. **A**: Schematic drawing of the organ of Corti (top panel) and representative images of IHCs and OHCs from adult mouse cochleae. **B**: Schematic drawing of the utricle (top panel) and confocal images of the utricle prepared from an adult mouse. HCs are stained with MYO6, SOX2 and DAPI. SOX2-positive cells include type II HCs and SCs underneath HCs. **C**: Representative images of type I and type II HCs from utricular and saccular maculae as well as crista ampullaris from adult mice. **D**, **E**: tSNE plots of distinct cell types detected in the adult CBA mouse cochlea (D) and utricle, saccule and crista. Different cell types are color-coded. **F**: Feature plots of the expression 6 marker genes in different HC populations. **G**: Dot plot heatmap of average expression and cellular detection rate of 26 representative marker genes in different HC types in cochleae and vestibular end organs. Abbreviations: IHC (inner HC); OHC (outer HC); SGN (spiral ganglion neuron); SC (Schwann cell)/SGC (satellite glial cell); DC (Deiters’ cell)/PC (pillar cell); IPhC (inner phalangeal cell)/IB (inner border cell); ISC (inner sulcus cell); HeC (Hensen’s cell); SpC (spindle cell)/RC (root cell); MC (marginal cell)/IC (intermediate cell); BaC (basal cell); MP (macrophage); RM (Reissner’s membrane); BC (B cell); TC (T cell); Gran (granulocyte); Mono (monocyte); RBC (red blood cell); OSC (outer sulcus cell); TBC (tympanic border cell). Type I HC (type I HC); Type II HC (type II HC); SESC (sensory epithelial SC); TEC (transitional epithelial cell); PTEC (peripheral transitional epithelial cell); ERC (epithelial roof cell). Neurons (vestibular neurons). For those cells whose definite identities cannot be annotated, the top expressed genes were used for identification and annotation. These cells include Tectb^+^, Mgp^+^, Coch^+^, Prl^+^, Otos^+^, Emilin2^+^/Cavin2^+^, Fxyd2^+^/Kcnk2^+^, Prtn3^+^/Ccl5^+^, and Ptgds^+^/Coch^+^.

Solitary cells were isolated from the whole basilar membrane (together with the organ of Corti, Fig. 1A) and vestibular end organs from 10-week-old CBA/J mice. Cells isolated from the cochlea include IHCs, OHCs, supporting cells (SCs), spiral ganglion neurons (SGNs), and other accessory cells. Some examples of individual IHCs and OHCs are shown in Fig. 1A. Cells isolated from vestibular sensory epithelia include type I HCs, type II HCs, SCs, vestibular neurons and accessory cells. Type II HCs and SCs express SOX2 (Fig. 1B). Thus, *Sox2*/SOX2 is a marker for these cell types in the vestibular sensory epithelia (Jan et al., 2021; Wilkerson et al., 2021). Some representative images of type I and type II HCs isolated from maculae of utricle and saccule as well as from crista ampullaris are presented in Fig. 1C.

To comprehensively assess the molecular profiles of inner ear HCs, we conducted scRNA-seq using cells isolated from the auditory and vestibular sensory epithelia. The cellular diversity in the cochlear and vestibular sensory epithelia was assessed by a t-distributed Stochastic Neighbor Embedding (t-SNE) analysis followed by cluster annotations based on the expression of known marker genes (Fig. 1D,E)(Burns et al., 2015; Jan et al., 2021; Ranum et al., 2019; Wilkerson et al., 2021). Upon cluster annotation, HC types were separated for the downstream analysis by assessing their generic and unique marker genes (Fig. 1F). For HC type, we aggregated the number of reads for each gene across all single cells to generate pseudo-bulk expression profiles (Table S1). Based on the expression of 26 core HC genes (Fig. 1G), 1,522 individual cells were identified as HCs, including 131 IHCs, 668 OHCs, 588 type I HCs, and 135 type II HCs for our downstream analysis.

### 1. Similarities among HC types

**Figure 2:**
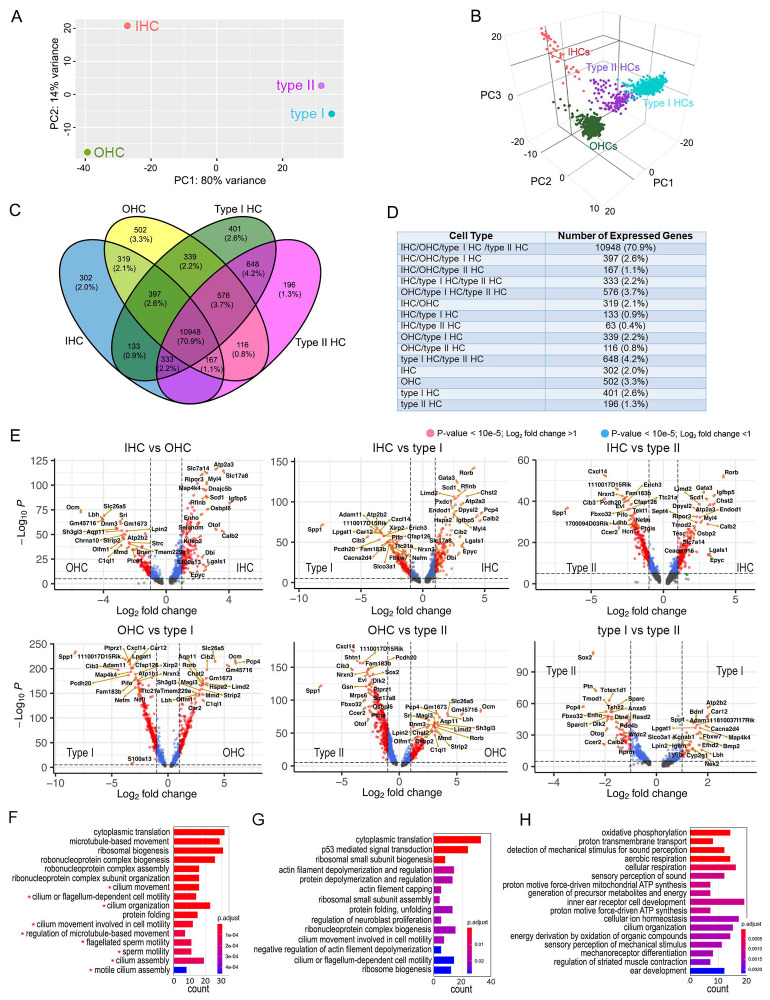
Similarity and difference among different HC types and biological processes enriched in cochlear and vestibular HCs. **A**: PCA plot showing similarity based on pseudo bulk RNA-seq data from four HC types. **B**: PCA plot showing similarity based on individual HC gene expression among the four HC types. **C**: Venn diagram depicting the number of expressed genes (RPKM > 0) in four HC types. **D**: The number and percentage of the total genes shared among two or more cell types or those uniquely expressed by a single cell type. **E**: Volcano plot showing differentially expressed genes between different HC types. Red dots indicate differentially expressed genes with P-value < 10^e^−5 and log_2_ fold change > 1. Only the top 20 differentially expressed genes are labeled. **F**: Biological processes enriched in vestibular HCs compared to cochlear HCs. Biological processes related to motile cilia are highlighted by red asterisks. **G**: Biological processes enriched in type I HCs compared to type II HCs. **H**: Biological processes enriched in type II HCs compared to type I HCs.

We utilized the pseudo-bulk and single-cell gene expression profiles to compare the four HC types. Since adult IHC and OHC transcriptomes have been compared extensively in the past (Li et al., 2018; Ranum et al., 2019), we focused our analyses on the differences between cochlear and vestibular HCs, as well as between type I and type II HCs. Principal component analysis (PCA) was used to denote variance across the four types of HCs. The first principal component (PC1) shows that the most dramatic differences are tissue-based; between cochlear and vestibular HCs (Fig. 2A). The distribution across PC2 indicates that the similarity between type I and type II HCs is greater than the similarity between IHCs and OHCs, suggesting more homogeneity among type I and type II HCs. Gene expression profiles of single HCs were also used to analyze similarities among four different HC types (Fig. 2B). Despite heterogeneity of individual HCs of each type, three-dimensional PCA visualization of single cells draws similar conclusions to the pseudo-bulk PCA, suggesting higher transcriptomic similarity between vestibular HCs in contrast to cochlear HCs.

### 2. Differentially expressed genes in HC types

Next, we examined the number of genes that are shared and unique among the four types of HCs based on pseudo-bulk expression profiles. Figs. 2C and D show the genes shared among two or more HC types, as well as those uniquely expressed by a single HC type. Approximately 71% of the detected genes are shared among all four types of HCs.

Although HCs share 71% of genes, the smaller proportion of genes unique in each HC population may underlie their specific biological identities. We performed a pairwise differentially expressed gene (DEG) analysis between HC types. DEGs were defined as those with an expression level above 0 and a minimum of 2-fold change (log2 ≥ 1) between the two cell populations with statistical significance of *p* ≤ 0.01. The volcano plots in Fig. 2E show differentially expressed genes between different HC types.

Comparison of IHCs and OHCs revealed the increase expression of 154 and 123 DEGs in IHCs and OHCs, respectively (Fig. 2E). This analysis revealed previously characterized DEGs in IHCs (e.g., *Otof, Slc17a8*) and OHCs (e.g., *Ocm, Slc26a5, Chrna10*) as well as genes whose functions have not yet been characterized, including *Atp2a3, Calb2, Dnajc5b, Ripor3* and *Scd1* in IHCs, and *Aqp11, Dnm3,* and *Sh3gl3* in OHCs.

Comparison between IHCs and type I HCs identified 174 DEGs in IHCs and 265 DEGs in type I HCs (Fig. 2E). Previously uncharacterized DEGs enriched in type I HCs such as *Adam11, Car12, Lpgat1, Spp1* and *Pcdh20* are related to cell-matrix interactions, mineralized matrix, calcification, and acid-base balance.

When IHCs are compared to type II HCs, 155 and 228 DEGs are found in IHCs and type II HCs, respectively (Fig. 2E). DEGs enriched in type II HCs include *Ccer2, Cib3, Cxcl14, Fbxo32, Ldhb, Nrxn3, Pcdh20* and *Spp1*. These genes play a role in the secretion of extracellular matrix protein, cell adhesion, phosphorylation-dependent ubiquitination, metabolism, and processes related to immunoregulation and inflammation.

When OHCs are compared to type I HCs, 180 and 289 DEGs are found in OHCs and type I HCs, respectively (Fig. 2E). *Adam11, Car12, Cib3, Lpgat1, Pcdh20,* and *Spp1* are highly enriched in type I HCs. Furthermore, the comparison between OHCs and type II HCs identified 197 DEGs in OHCs and 317 DEGs in type II HCs. While most DEGs overlapped with those identified in the IHC and type II HC comparison, this analysis revealed additional type II HC DEGs such as *Evl, Gsn, Fam183b, Mrps6*, and *Nrxn3*. Comparison of type I and type II HCs revealed the enrichment of 35 DEGs in type I HCs and 59 DEGs in type II HCs. Newly identified markers for type II HCs include *Dlk2, Dtna, Fbxo32, Otog, Pcp4, Ptn, Sparc, Sparcl1, Tctex1,* and *Tmod1* whereas the top DEGs in type I HCs included *Adam11, Atp2b2, Bdnf, Bmp2, Cacna2d4, Car12, Kcnab1, Lpgat1* and *Spp1*.

Since the molecular properties of vestibular HCs are less known compared to cochlear HCs, we performed a gene ontology (GO) and overrepresentation analysis to examine biological processes enriched in vestibular HCs compared to cochlear HCs. The comparison of processes enriched in vestibular HCs revealed GO terms related to cilium organization and microtubule-based cilium motility (highlighted by red asterisks in Fig. 2F). We also compared the biological processes enriched in type I and type II HCs. This comparison revealed the enrichment of processes related to oxidative phosphorylation, proton transmembrane transport, aerobic and cellular respiration in type I HCs (Fig. 2G), while the enriched processes in type II HCs are related to p53-mediated signal transduction and actin filament depolymerization and regulation (Fig. 2H).

### 3. Marker genes for different HC types and genes related to HC specialization

**Figure 3:**
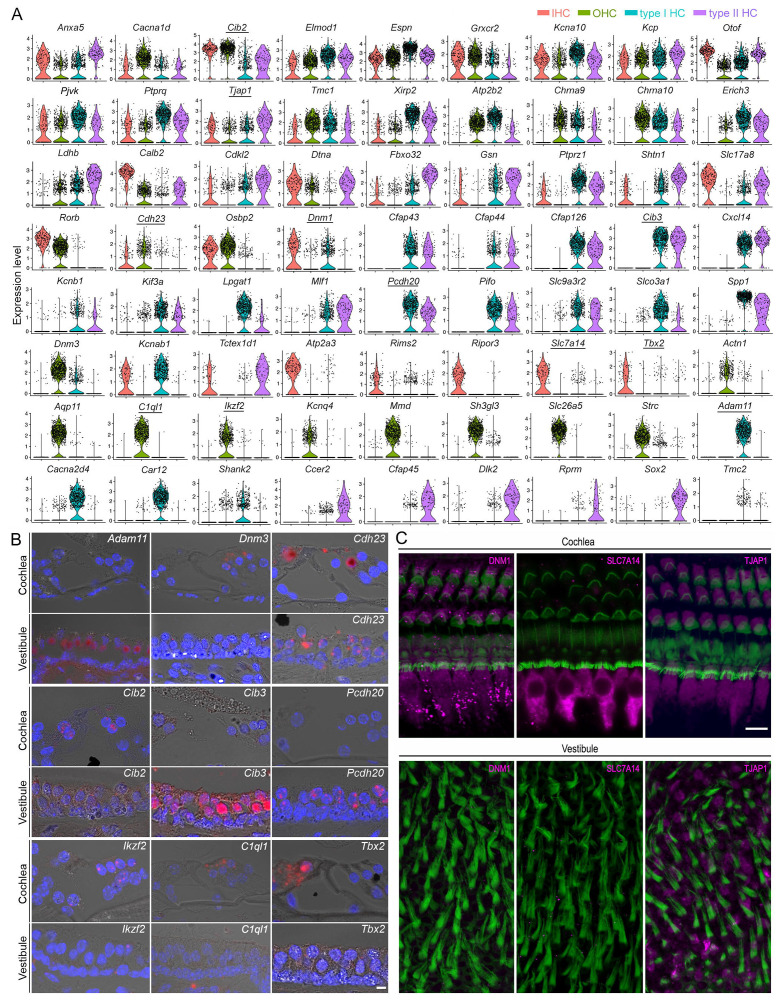
Shared and unique genes expressed in cochlear and vestibular HCs. **A**: Violin plots of the expression 72 genes in four different HC types. **B**: Validation of differential expression of 9 genes (with underline in **A**) in cochlear and vestibular HCs in thin section. Bar: 5 μm for all images in B. **C**: Confocal images of expression of DNM1, SLC7A14, and TJAP1 in cochlear and vestibular HCs. Bar: 10 μm for all images in C.

Previous studies have characterized some marker genes in different HC types. We assessed the expression of previously characterized and newly identified HC genes across all four HC types (Fig. 3A). Genes shared among all four HC types include *Cib2, Tmc1,* and *Espn*. We also identified genes not known to be expressed in all HC types. An example is *Tjap1,* which encodes a tight junction-associated protein. Some genes were found to be enriched in a tissue-based manner. For example, *Cdh23, Rorb* and *Osbp2* are more highly expressed in cochlear HCs than in vestibular HCs; meanwhile, genes such as *Ldhb, Fbxo32, and Gsn* are enriched in vestibular HCs but only weakly expressed in cochlear HCs. We identified several genes that are only expressed in vestibular HCs but not in cochlear HCs (such as *Cfap43, Cfap44, Cfap126, Cib3, Cxcl14, Kcnb1, Kif3, Lpgat1, Mlf1, Pcdh20, Pifo, Slc9a3r2, Slco3a1, Tmc2* and *Sppl1*), which could potentially be used as markers for vestibular HCs. Moreover, *Adam11, Cacna2d4, Car12,* and *Shank2* are found to be only expressed in type I HCs, while *Ccer2, Cfap45, Dlk2,* and *Rprm* are only expressed in type II HCs. Our analysis also revealed new marker genes for IHCs (*Atp2a3, Rims2, Ripor3*) and OHCs (*Aqp11, Mmd* and *Sh3gl3*). *Cfap43, Cfap44, Cfap45, Cfap126, Kif3, Mlf1* and *Pifo,* differentially expressed in vestibular HCs, are all associated with kinocilium structure and function.

We utilized single-molecule fluorescent *in situ* hybridization (smFISH) and immunostaining techniques to validate the expression of 12 genes in HCs (Fig. 3B, C). The expression patterns of these genes and proteins were highly consistent with our observations from scRNA-seq analysis. Overall, our results identified novel marker genes and previously unidentified expression patterns among the four HC types.

**Figure 4:**
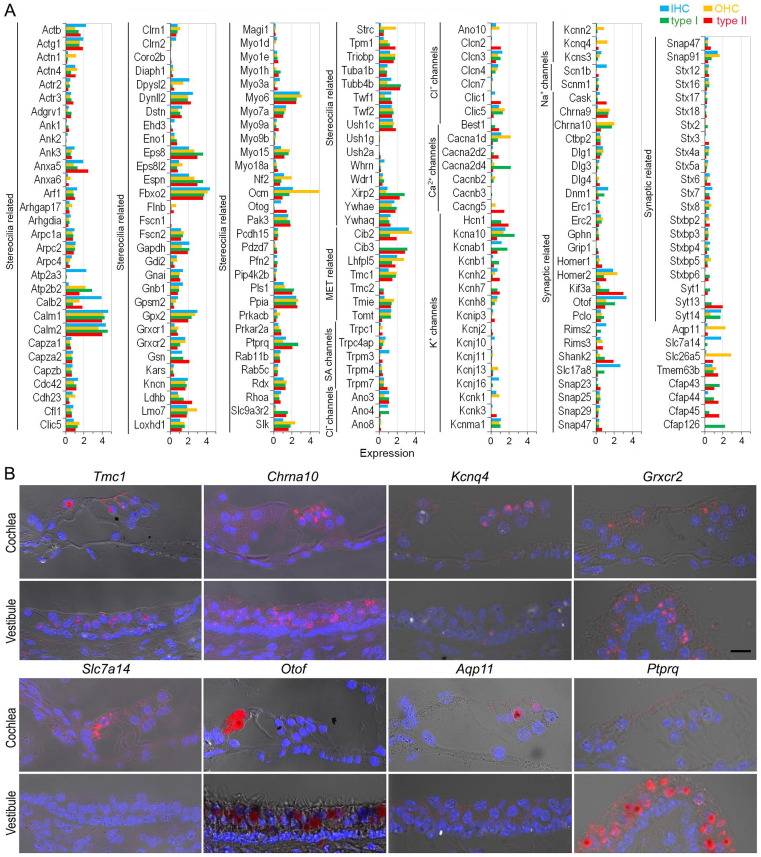
Expression of genes related to HC specialization. **A**: Heatmap showing expression of genes related to stereocilia bundles, mechanotransduction, ion channels and synaptic structure. **B**: Validation of gene expression using smFISH. Bar: 10 μm.

Next, we focused our analysis on evaluating the genes associated with HC function. We compared the expression of 208 genes related to HC specialization, including stereocilia and apparatus for mechanotransduction, ion channels, and synapses (Fig. 4A). Among the genes associated with stereocilia and mechanotransduction apparatus(Shin et al., 2013), *Calm1, Calm2, Eps8, Espn, Fbxo2, Dynll2, Ush1c, Ywhae, Tmc1, Tmie, Cdh23, Pcdh15, Ank1, Ank3,* and *Lhfpl5* were highly expressed in all four HC populations. Others were highly expressed in only one or two HC populations. *Atp2a3, Calb2,* and *Dpysl2* were highly expressed in IHCs, while *Lmo7, Ocm,* and *Strc* were highly expressed in OHCs. Moreover, expression of *Dpysl2, Cdh23,* and *Cib2* was higher in cochlear HCs than in vestibular HCs, while *Xirp2, Pls1, Slc9a3r2,* and *Tubb4b* were more highly expressed in vestibular HCs than in cochlear HCs. Some genes were uniquely expressed in either cochlear or vestibular HCs. For example, *Cib3* and *Tmc2* are expressed in vestibular HCs but not in cochlear HCs.

All HCs possess ion channels. Our analysis detected several genes related to stretch-activated ion channels, such as *Trpc1* and *Trpm4*. Genes for Cl^−^ and Na^+^ channels were expressed. For Ca^2+^ and K^+^ channels, *Cacna1d and Kcna10* were expressed in all four HC types with varying levels of expression. In cochlear HCs, *Cacna1d, Kcna10, Kcnab1, Kcnj16 and Kcnma1* were expressed in IHCs, whereas *Cacna1d, Kcna10, Kcnk1, Kcnma1, Kcnn2* and *Kcnq4* were expressed in OHCs. In the vestibular HCs, *Cacna2d4, Kcna10, Kcnab1,* and *Kcnma1* showed relatively high expression in type I HCs whereas type II HCs indicated a relatively high expression of *Cacna2d4, Cacng5, Kcna10, Kcnb1, Kcnh2,* and *Kcnh7*. *Best1, Clic, Hcn1,* and *Kcnh7* were only expressed in vestibular HCs.

Next, we examined the genes related to synapses (Fig. 4A). Our results indicated expression of *Ctpb2, Dlg1*, *Homer2, Otof, Pclo*, and *Snap91* in all HCs at varying levels. A higher expression of *Dnm1, Otof, Rims2, Slc17a8, Snap91,* and *Stx7* was seen in IHCs while *Dlg1, Dnm3*, *Snap9, Chrna9,* and *Chrna10* showed higher expression in OHCs. Some of the highly expressed genes in type I HCs include *Dnm1, Kif3a, Pclo, Shank2, Slc17a8, Syt13, Syt14, Chrna9,* and *Chrna10,* whereas type II HCs showed relatively higher expression of *Kif3a, Otof, Shank2, Stx7,* and *Syt13*. We employed smFISH to validate the expression of 8 additional genes across the four HC types. The expression patterns shown in Fig. 4B are consistent with our analysis (Fig. 4A).

### 4. Gene signatures of primary cilia in cochlear and vestibular HCs

**Figure 5.**
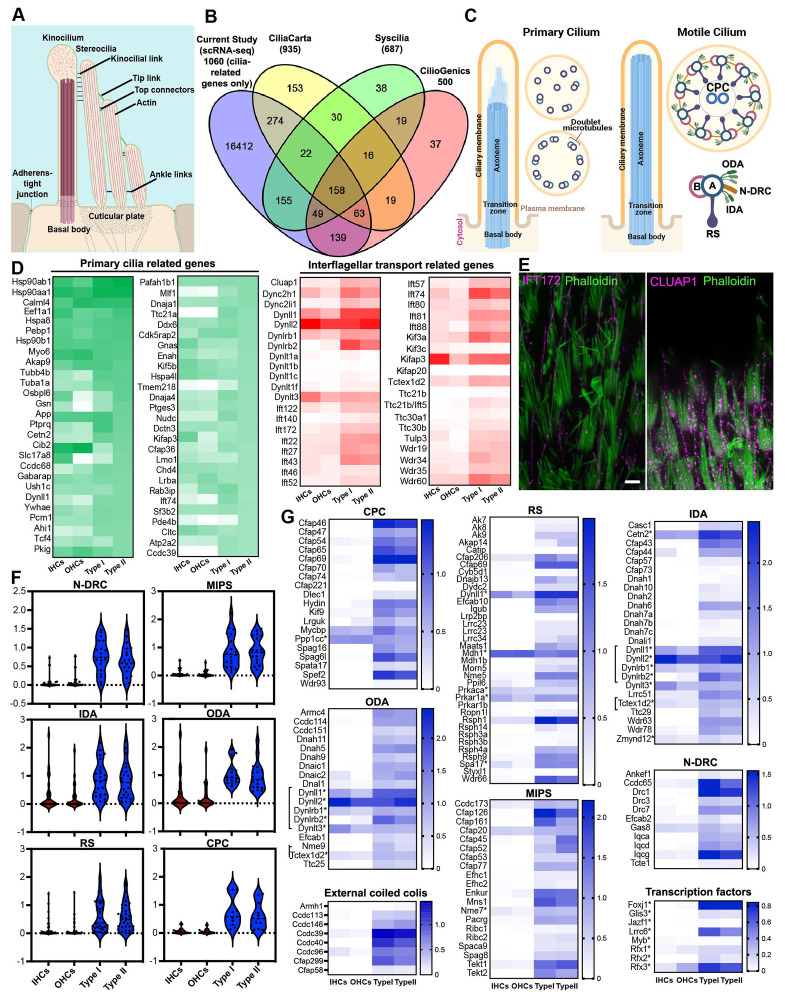
Cilia-related genes detected in cochlear and vestibular HCs. **A:** Schematic illustration of HC hair bundle (adapted from Schwander et al.(Schwander et al., 2010)). **B**: Venn diagram illustrating the number of genes in each database and the cilia-related genes detected in HC transcriptomes. **C**: Schematic illustration of primary and motile cilia, highlighting 9+0 or 9+2 arrangement of microtubules for primary and motile cilia, respectively: radial spokes (RS), central pair complex (CPC), nexin–dynein regulatory complex (N-DRC), microtubule inner proteins (MIPs), inner and outer dynein arms (IDA and ODA). **D**: Expression of top 50 cilia-related genes and genes related to IFT in the four types of HCs. **E**: Immunostaining of IFT172 and CLUAP1 expression in vestibular kinocilia. Bar: 5 μm. **F**: Violin plots showing aggregated expression of genes associated with 96-nm repeat. **G**: Heatmaps showing the comparison of gene expression related to motile-cilia machinery: radial spokes (RS), central pair complex (CPC), nexin–dynein regulatory complex (N-DRC), microtubule inner proteins (MIPs), inner and outer dynein arms (IDA and ODA), external coiled coils, and motile cilia transcriptional regulators in cochlear and vestibular HCs.

A key morphological feature in the hair bundle of vestibular HCs is the presence of kinocilium, which has been regarded as a type of specialized primary cilia. Since our analysis revealed an enrichment of cilium-related GO terms (Fig. 2F) and axonemal genes such as *Cfap43, Cfap44, Cfap45, Cfap126, Kif3, Plf1,* and *Tubb4b* (Fig. 3A) in vestibular HCs, we sought to investigate the composition and nature of the kinocilium (Fig. 5A). Proteomics-based approaches have contributed to the development of cilia-associated protein databases. We utilized several well-established databases, including CiliaCarta (Dam et al., 2019), the SYSCILIA gold standard (SCGSv2)(Vasquez et al., 2021), and CilioGenics(Pir et al., 2024) to compile a list of ~1,000 cilia-related genes. We noted a significant overlap of these genes with our transcriptome (Fig. 5B). GO analysis of the overlapping genes revealed enrichment of cellular component and biological process terms primarily related to cilia organization, assembly, maintenance, intracellular transport, and microtubule dynamics particularly associated with motile cilia. Additionally, molecular function analysis highlights associations with motor activity, BBSome binding, and dynein chain binding (Fig. S1).

Primary cilium is a sensory organelle that responds to and transmits external signals to the cell's interior. Structurally, primary cilia are characterized by the presence of nine microtubule doublets (MTDs) encircling the shaft, which transition into a disorganized structure distally (Fig. 5C). We first examined the expression of primary cilia-related genes in vestibular and cochlear HCs. Among approximately 420 primary cilia-related genes/proteins (Dam et al., 2019; Pir et al., 2024; Vasquez et al., 2021), we detected the expression of 410 genes in at least one type of HC. Fig. 5D shows the top 50 abundantly expressed primary cilia-related genes in type II HCs compared to the other three HC types. Most of these genes were detected in all four HC types except a few genes, such as *Mlf1, Ttc21a,* and *Tmem218,* that were weakly or not expressed in cochlear HCs.

Primary cilia are enriched in receptors and effectors for key pathways, including GPCR, cAMP, Ca^2+^, RTK, TGF-β, MAPK, TOR, BMP, Wnt, Notch, and Rho signaling (Anvarian et al., 2019), localized to the ciliary shaft, transition zone, and BBSome (Hansen et al., 2024). We analyzed the expression of these genes in HCs and found higher expression levels of these pathway-related genes in vestibular HCs than in cochlear HCs (Fig. S2).

Intraflagellar transport (IFT) involves anterograde and retrograde transport of molecules along the axoneme of cilia, facilitating the transport of components between the ciliary base and tip (Ma et al., 2023). IFT is essential for the proper assembly and maintenance of both primary and motile cilia. Thus, we assessed the expression of IFT-associated genes in HCs. Most of the genes are enriched in vestibular HCs compared to cochlear HCs (Fig. 5D). Immunostaining confirmed the expression of IFT172 and CLUAP1 in the kinocilia of vestibular HCs (Fig. 5E).

### 5. Gene signatures of motile cilia in vestibular HCs

Motile cilia are highly conserved organelles across different organisms and tissues, although they exhibit organism- and tissue-specific adaptations (Leung et al., 2025). Recent advances using proteomics of isolated motile cilia from various ciliated tissues have enabled the profiling of genes associated with motile cilia. To explore whether the kinocilium possesses a molecular composition characteristic of motile cilia, we compared our HC transcriptomes with multi-tissue proteomics datasets derived from different organisms, including human, bovine, porcine, and murine, as well as diverse motile ciliary tissues such as sperm, oviduct, ventricle, and trachea (Leung et al., 2025) (Fig. S3). Our analysis revealed a significant overlap, particularly among axonemal components of motile cilia, with strong enrichment in vestibular HCs compared to cochlear HCs. The axoneme of motile cilia and flagella is a cylindrical structure harboring a canonical “9+2” arrangement, where nine microtubule doublets surround two microtubule singlets in the center (Fig. 5C). Altogether, the axoneme machinery consists of nine DMTs, two rows of inner and outer dynein arms (IDAs and ODAs), nexin-dynein regulatory complex (N-DRC), two singlet central pair complex (CPC), three radial spokes (RSs), microtubule inner proteins (MIPs), and external coiled-coil regions. The motility unit is arranged in a 96-nm repeat module along the CPC. Recent cryo-EM and cryo-electron tomography (cryo-ET) studies have provided a more comprehensive identification of this module repeat (Chen et al., 2023; Leung et al., 2025; Walton et al., 2023). To understand the molecular composition and function of kinocilia, we focused our analysis on the expression of genes associated with the structure of motile cilia. First, we aggregated the genes related to each component of the motile cilia structure and assessed the expression of these genes among the HC types. We noted a robust expression of motile cilia-related gene signatures in vestibular HCs, while cochlear HCs expressed little to none (Fig. 5F). Next, we further assessed the expression of the key genes related to motile cilia machinery (Fig. 5G), including the 96-nm module and CPC, based on the current known localization from biochemistry and proteomics.

The 96-nm axonemal repeat of mammalian epithelial cilia contains proteins encoded by 128 genes (Gui et al., 2021). We found that 112 out of 128 genes related to axonemal repeat were expressed in adult vestibular HCs, while only ~15% of those were detected in cochlear HCs. Genes encoding axonemal dynein (IDAs and ODAs), such as *Dnah5* and *Dnah6*, as well as radial spoke components (*Wdr66, Cfap206, Cfap61, Iqub*), N-DRC components (*Drc1, Iqca*), and MIPs (*Cfap126, Wdr63*) were predominantly expressed in vestibular HCs with little to no expression in cochlear HCs (Fig. 5G). Axonemal CCDC39 and CCDC40, which form external coiled-coil regions, are the molecular rulers that organize the axonemal structure in the 96-nm repeating interactome and are required for the assembly of IDAs and N-DRC for ciliary motility (Becker-Heck et al., 2011; Merveille et al., 2011; Oda et al., 2014). Our results indicate a high expression of *Ccdc39* and *Ccdc40* in vestibular HCs, whereas little to no expression was observed in cochlear HCs.

RFX and FOXJ1 transcription factor families are key regulators of ciliome gene activation, with RFX controlling genes in both motile and non-motile cilia, while FOXJ1 specifically governs motile cilia formation (Choksi et al., 2014). Our data indicated that *Foxj1* was moderately expressed in vestibular HCs but only weakly expressed in cochlear HCs (Fig. 5G). Other genes that regulate motile cilia formation, including *Lrrc6,* were also expressed at high to moderate levels in vestibular HCs compared to cochlear HCs. We also found a strong enrichment of transcriptional targets associated with vestibular HCs, particularly those involved in motile cilia programming and maintenance. Thus, the expression of these transcription factors, including *Foxj1* may suggest that their expression is necessary for the maintenance of kinocilia in adult vestibular HCs. Furthermore, analysis of a published ATAC-seq dataset (Jen et al., 2019) from adult mouse vestibular tissue revealed increased chromatin accessibility in the promoter regions of genes associated with motile cilia machinery (Fig. S4), suggesting elevated transcriptional activity in vestibular HCs. These findings are consistent with our observations from scRNA-seq datasets.

**Figure 6:**
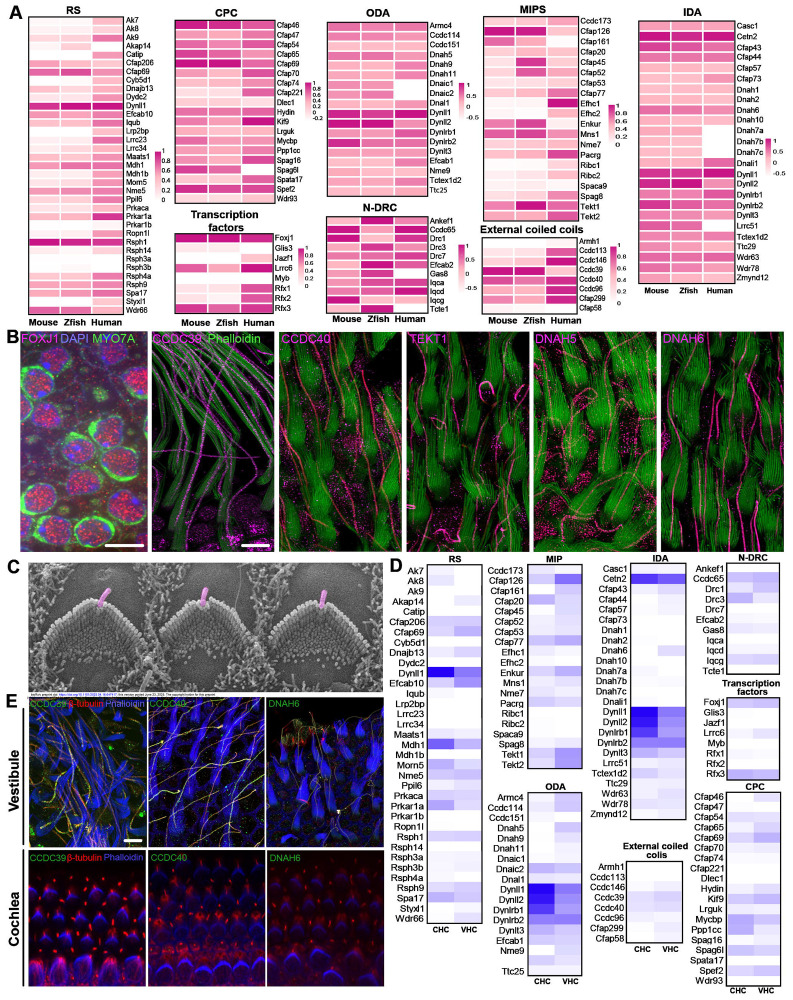
Expression of motile cilia-related genes/proteins in the vestibular HCs. **A:** Expression of motile cilia-related genes in mouse, zebrafish, and human vestibular HCs. Mouse gene nomenclature is used in the heatmaps. **B:** Confocal images of the expression of key motile cilia-related proteins. Scale bars represent 5 μm. **C**: SEM micrograph of hair bundles of OHCs from P2 cochlea. Kinocilia (in magenta) are still present at this age. **D:** Comparison of expression of motile cilia-related genes between P2 cochlear and vestibular HCs. **E:** Confocal images of expression of CCDC39, CCDC40 and DNAH6 in P2 vestibular and cochlear HCs. CCDC39, CCDC40, and DNAH6 were not expressed in cochlear HCs at P2. Bar: 5 μm.

Next, we examined whether the expression of these genes is conserved in vestibular HCs across different species. We obtained the orthologs of axoneme-related genes in adult zebrafish inner ear HCs and human vestibular HCs using published datasets (Barta et al., 2018; Wang et al., 2024). Fig. 6A shows the expression of these genes in adult mouse, zebrafish, and human vestibular HCs. While the expression level varies, the majority of these genes are expressed across species. The exceptions are *Dnah3* and *Dnah12*, which are expressed in zebrafish HCs but not in mammalian vestibular HCs.

We conducted immunostaining and high-resolution confocal imaging to validate the expression of key motile cilia markers in the kinocilia. As indicated in Fig. 6B, FOXJ1 is expressed in the nucleus of vestibular HCs, while CCDC39, CCDC40, TEKT1, DNAH5, and DNAH6 are expressed in the kinocilia. Collectively, our findings provide evidence corroborating the presence of motile cilium machinery in the kinocilia of vestibular HCs.

Since nascent cochlear HCs possess kinocilia (Fig. 6C), we investigated whether cochlear HCs express motile cilium-related genes by utilizing published P2 cochlear and vestibular HC transcriptomes (Burns et al., 2015). Assessment of expression of genes related to motile cilia machinery in the nascent vestibular and cochlear HCs revealed a less drastic difference between neonatal cochlear and vestibular HCs than observed between adult HCs (Fig. 6D). However, we did note the absence of some key genes such as *Dnah6, Dnah5,* and *Wdr66* in the P2 cochlea. Immunostaining confirmed the lack of expression of CCDC39, CCDC40, and DNAH6 in cochlear HCs at the protein level while they were expressed in kinocilia of vestibular HCs at P2 (Fig. 6E). Thus, our analysis shows that the molecular composition of kinocilia is different between neonatal cochlear and vestibular HCs and that the kinocilium of neonatal cochlear HCs does not possess signatures of motile cilia.

### 6. Vestibular kinocilia exhibit hybrid morphological features of primary and motile cilia

**Figure 7:**
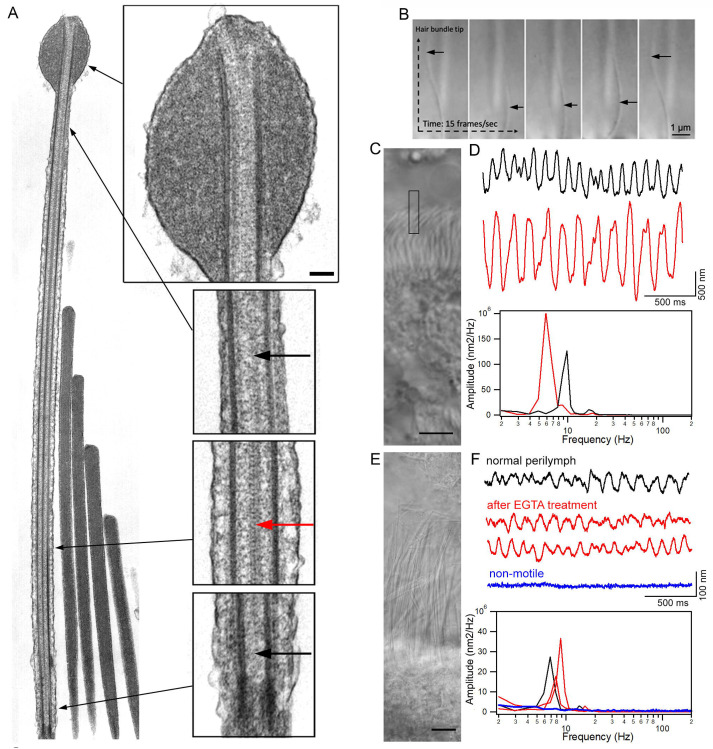
Kinocilia morphology and motility. **A**: TEM images of stereocilia and kinocilium from bullfrog crista HCs. Different regions of the kinocilium in higher magnification are also shown. Long black arrows indicate where the magnified images were taken. Bar: 250 nm. Red arrow indicates two central microtubule singlets. Short black arrows mark absence of central microtubule singlets in the distal regions near the tip of kinocilium and transition zone. **B**: Images captured from *in vitro* live imaging of kinocilium and bundle motion of a bullfrog crista HC. The images were captured with speed of 15 frames per second. Black arrows indicate kinocilium. **C**: DIC image of cilia from mouse middle ear. Black frame marks where the motion was measured. Bar: 5 μm. **D**: Representative waveforms of spontaneous ciliary motion from middle ear tissue. The FFT analysis of cilia motion is also shown. **E:** DIC image of hair bundles from mouse crista HCs. Bar: 10 μm. **F**: Three representative waveforms of spontaneous motion of hair bundles. The response in blue shows measurements taken from a hair bundle with no spontaneous motility. Time scale in F also applies to D. FFT analysis of bundle motion is also shown. Response waveforms and spectra are color-coded and -matched.

TEM studies have characterized the ultrastructure of kinocilia across species, revealing evidence of complex and regionally specialized organization (Kikuchi et al., 1989; Nagel et al., 2014; O’Donnell and Zheng, 2022). TEM images, including longitudinal sections of frog vestibular hair bundles (Fig. 7A) highlight a distinct zonal architecture along the kinocilium axis. At the distal tip, a prominent kinociliary bulb is observed, while the base anchors the axoneme within the cuticular plate. Two pairs of microtubule doublets span the full length of the kinocilium. The central pair of singlet microtubules, typical of motile cilia, is maintained throughout most of the shaft but disappears in the distal and transitional zones. This configuration results in a dynamic shift from a canonical 9+2 arrangement centrally to a 9+0 pattern at both the base and tip. These observations underscore the heterogeneous and hybrid nature of vestibular kinocilia, integrating structural features of both primary and motile cilia. Such duality likely supports their unique functional role at the intersection of signal transduction and mechanosensation.

### 7. Evidence that vestibular kinocilia exhibit motility

Vestibular kinocilia are traditionally regarded as non-motile, lacking the rhythmic beating characteristic of respiratory cilia. Using acute preparations of bullfrog semicircular canal sensory epithelia (cristae), we observed robust spontaneous kinociliary motility in some HCs (Fig. 7B; Videos S1 and S2). This motility exhibited the characteristic flagella/cilia beating pattern and occurred at a frequency of approximately 10 Hz at the room temperature. The observed displacements were sufficiently large to induce deflection of the entire hair bundle, indicating it can generate forces to influence hair bundle dynamics. Interestingly, this phenomenon was detected in only ~1-5% of crista HCs, likely due to variable preservation of HC and kinociliary integrity *in vitro* in acutely dissected tissue. Kinocilia are not only tightly connected to stereocilia but are also tightly connected to the overlaying gelatinous membrane (Eatock et al., 1998; Leibovici et al., 2005), an extracellular matrix required for physiological mechanotransduction. While motility is not experimentally observed in all or the majority of HCs in the excised vestibular sensory epithelium, these findings suggest that vestibular kinocilia are capable of active movement and their motility could be triggered under more physiological conditions, challenging the strict classification of these structures as non-motile.

Next, we explored whether the kinocilia of vestibular HCs from adult mice are motile by utilizing the photodiode technique to detect bundle motion (Jia and He, 2005). This technique can detect motion in the 10-nm range for synchronized signals after averaging. Since spontaneous motions are not synchronized and cannot be averaged to improve signal-to-noise ratio, we captured the responses in the time domain and averaged them in the frequency domain after a fast Fourier transform. This allows us to detect unsynchronized cilia motion even if the signal is close to the noise level at the time domain. We first used the cilia in the epithelial lining of the Eustachian tube, which is an extension of airway epithelia, as a positive control for motility measurement. Fig. 7C shows an image of airway cilia using differential interference contrast optics. We measured motion of the top segment of cilia. Fig. 7D shows two representative waveforms of airway cilia beat with a magnitude between 700 and 1500 nm. The two responses shown in Fig. 7D have main frequency components at 6 to 9 Hz. Next, we measured the movement of the top segment of the hair bundle from mouse crista ampullaris (Fig. 7E). Since the kinocilium is tightly attached to the stereocilia bundle, we measured the whole bundle's motion due to the difficulty of taking measurements from a single kinocilium. The waveform (black trace in Fig. 7F) was obtained from crista HCs bathed in perilymph-like solution (L-15 medium) with 2 mM of Ca^2+^. When the motion was analyzed in the frequency domain, the peak at ~7 Hz was evident (Fig. 7F). To rule out the possibility that the bundle motion is driven by the mechanotransduction-related activity (Benser et al., 1996; Martin et al., 2003), we treated the crista ampullaris in Ca^2+^-free medium with EGTA for 2 minutes to break the tip-link (Jia et al., 2007; Ricci et al., 2003). Spontaneous bundle motion was still detected (two red traces in Fig. 7F), suggesting that the motion is independent of the transduction channel activity. We measured spontaneous bundle motions from 52 crista HCs from six mice. Spontaneous motion was only detected in eight HCs while the majority of HCs did not display detectable bundle motion. An example of a lack of response is shown in Fig. 7F (blue trace). Although we were unable to determine the type of HCs that exhibited kinocilium motility, it is conceivable that the kinocilia of type I and type II HCs are motile since the genes related to motile cilia were detected in both types of vestibular HCs. We note the kinocilia motion of mouse crista HCs was substantially smaller than that of airway cilia (Fig. 7D) and bullfrog crista HCs.

### 8. Predicted model of the 96-nm modular repeat in adult vestibular kinocilia

**Figure 8:**
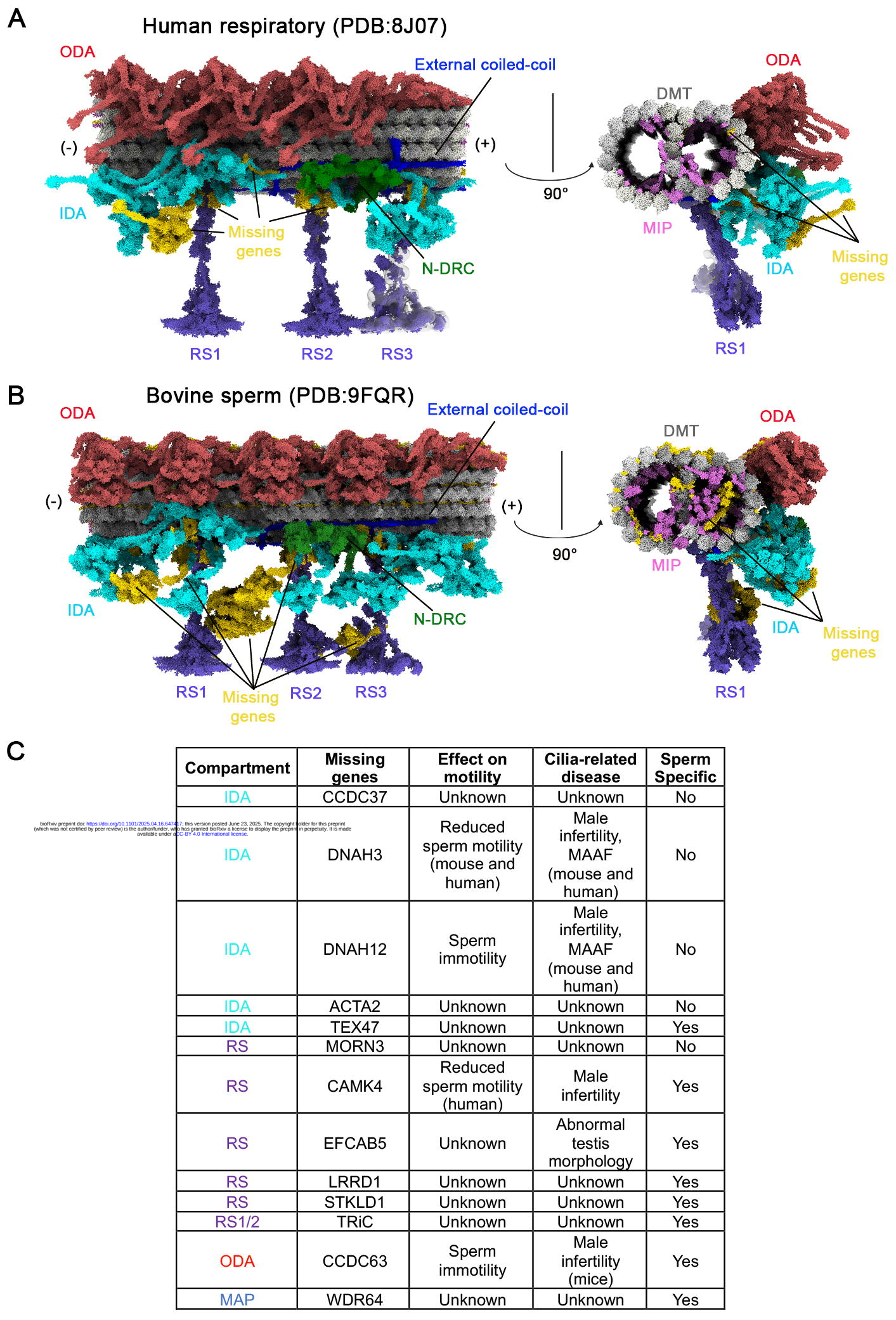
Predicted models of the molecular architecture of 96 nm axonemal repeat of vestibular kinocilia. **A-B**: Longitudinal and cross-sectional views of the doublet microtubule and associated structure in 96-nm repeat, derived from combining cryo-EM data and single-cell transcriptomic analysis from human respiratory (A) and bovine sperm flagella (B). Key axonemal motile-machinery components are color-coded: ODA (Indian red), IDA (cyan), N-DRC (green), MIPs (orchid), RS (purple), and external coiled-coils (blue). Radial spoke 3 (RS3) has not been resolved to atomic resolution, but its shorter form (RS3s) is depicted. Doublet microtubules (DMT) are represented in gray. Regions highlighted in gold indicate the absence of corresponding transcripts in our mouse transcriptomic data. **C**: Genes which are not detected in mouse and human vestibular HC transcriptomes and related to motility-relevant compartments are listed in the table. The roles of these genes in the 96-nm repeat module and cilia motility and ciliopathy are also included.

Since kinocilium motility in mouse vestibular HCs is substantially smaller than the motility of airway cilia, we investigated the structural basis underlying comparatively reduced motility of kinocilia. This diminished motility may result from differences in the molecular composition and organization of the axonemal machinery, particularly the 96-nm modular repeat that houses key dynein motors and regulatory complexes. Recent advances in structure prediction powered by artificial intelligence and cryo-electron microscopy (cryo-EM) have facilitated the generation of highly conserved atomic models of the 96-nm axonemal repeat from human respiratory cilia and bovine sperm flagella (Chen et al., 2023; Leung et al., 2025; Walton et al., 2023). We applied our axonemal gene dataset to these atomic models to predict the molecular architecture of the 96-nm repeat in vestibular kinocilia. By mapping the expression of known axonemal components onto the structural frameworks derived from human respiratory (PDB: 8J07) (Walton et al., 2023) and bovine sperm (PDB: 9FQR)(Leung et al., 2025) axonemes, we generated two composite models that reflect the unique molecular composition of the vestibular kinocilium. These predicted structures are shown in Figs. 8A and 8B.

We chose the human respiratory 96-nm axonemal structure as our reference because it best reflects vestibular kinocilia and provides a more comprehensive representation of axonemal components than the sperm model, which shows more missing elements (highlighted in gold in Figs. 8A and B). Using a curated reference gene list derived from the human respiratory 96-nm axonemal structure, we mapped vestibular HC gene expression and identified transcripts for all 18 ODA genes and their docking complex components, all 11 N-DRC genes, and all 7 MAPs, along with 36 of 37 radial spoke genes and 19 of 23 IDA-related genes. Additionally, 20 of the 31 genes encoding MIPs, which are known to stabilize the microtubule doublets, were also expressed (Fig. 5G). In contrast, vestibular HCs lacked the expression of several sperm-specific genes from distinct compartments of 96-nm repeat (Leung et al., 2025), including TRiC chaperonin subunits (Brown et al., 2025; X. Meng et al., 2024), CAMK4, EFACB5, LRRD1, STKLD1, CCDC63, WDR64, and several MIPs (Fig. S5)^26^.

Based on our models, the absence of *Dnah3* and *Dnah12*, along with *Acta2*, is predicted to result in the loss of two of the six single-headed IDA components (highlighted in gold). *Cfap100*, which forms the modifier of inner arms (MIA) complex with *Cfap73* and contributes to tethering the double-headed IDAf (inner dynein arm f), is missing in the model, whereas *Cfap73* and the remaining IDAf components are present. Notably, IDAf has multiple attachment points, and MIA is not essential for docking IDAf to the DMTs (Yamamoto et al., 2013). Overall, 4 of the 23 IDA-related genes were not expressed in vestibular HCs; nonetheless, we predict that the IDA structure is reduced but not entirely absent, as DNAH6 remains localized in vestibular kinocilia (Fig. 5B). In addition to the missing IDA components, we identified 11 unexpressed genes associated with MIPs, whose absence is predicted to result in reduced MIP density in the models (Figs. 8A and B, highlighted in orchid and gold in cross-sectional views). Unlike other axonemal structures, MIPs exhibit greater variability across species, which may account for their lineage-specific absence in vestibular kinocilia (Fig. 8)(Andersen et al., 2024; Tai et al., 2023; Xia et al., 2025). The missing genes in our HC datasets and their roles in axonemal complex, cilium motility and ciliopathy are listed in Figs. 8C and S5. Based on our predicted models, we speculate that the absence of *Dnah3* and *Dnah12* plays a major role in limiting kinocilium motility in mouse vestibular HCs, contributing to the smaller movements compared to motile cilia.

## DISCUSSION

This is the first study to compare transcriptomes among four types of HCs from the adult mouse inner ear and to characterize the unique hybrid property of the kinocilia. We observed several differences in gene signatures related to HC specialization, which may underlie distinct properties of mechanotransduction, membrane conductance, and synaptic transmission seen among the four different HC types. Differential expressions also explain why loss of function of a gene such as *Tmc1, Cib2,* or *Cib3* leads to differential auditory and vestibular phenotypes in mouse models and humans. Our dataset is expected to serve not only as a highly valuable resource for unraveling the molecular mechanisms of the biological properties of HCs but also for assisting the auditory and vestibular research community in identifying and exploring the functions of disease-related genes.

Since biological processes enriched in vestibular HCs are related to cilia and cilium motility, we focused our analyses on kinocilia. The kinocilia has long been regarded as the primary cilium. However, the molecular composition and structural organization of kinocilia are largely unexplored. Our study suggests that kinocilia serve dual roles as both primary and motile cilia. Primary cilia are a major hub in receiving and transmitting signals from the environment. A recent study has demonstrated the expression of proteins related to transduction pathways and receptors in primary cilia using spatial proteomics (Hansen et al., 2024). We showed that vestibular and cochlear HCs expressed genes encoding proteins for signal transduction pathways and receptors. Although the localization of those proteins in HC kinocilia needs further validation, our analysis highlights the expression of diverse primary cilia signaling genes shared among different HC types, emphasizing their conserved and potentially specialized roles in cellular signaling and ciliary function.

The tip of motile cilia is a region with specialized proteins (Legal et al., 2023). In multi-ciliated cells, CCDC33 and CCDC78 are found at the very end of the cilium and help organize other proteins like SPEF1, CEP104, and EB3/MAPRE3 (Hong et al., 2025; Legal et al., 2024, 2023). We found expressions of all the genes encoding these proteins except *Ccdc78*. Although we did not study the tip of the kinocilium, its bulb shape suggests it may also contain specialized proteins. In bullfrog HCs, the kinocilial bulb binds to the overlaying otoconial membrane (Jaeger et al., 1994; Kachar et al., 1990), and shows strong labeling for β-tubulin and cadherin 23 (Jaeger et al., 1994; Kachar et al., 1990; Lagziel et al., 2005), and a recent study showed that *saxo2* overexpression causes bulbed zebrafish kinocilia with Saxo2 accumulating at the tip (Erickson et al., 2023). These bulbed tips may reflect specialized regulation of ciliary cap proteins (Legal et al., 2023) that help organize or stabilize the plus ends of axonemal microtubules—an area that remains to be explored in kinocilia.

The most important and novel finding of our study is that adult vestibular HCs express genes related to the 96-nm axonemal repeat complex, a hallmark structural component of motile cilia. Orthologous axonemal-related genes are identified in zebrafish and human vestibular HCs, underscoring the evolutionary conservation of the molecular composition of the complex in vestibular HCs across vertebrates. Furthermore, we observed robust spontaneous kinocilia motility in bullfrog crista HCs and small spontaneous bundle motion in mouse crista HCs. The movement is clearly not driven by mechanisms associated with the mechanotransduction apparatus, as the kinocilium itself displayed an active flagellar-like movement in bullfrog crista HCs. In mouse crista HCs, spontaneous motion was still present after breaking the tip links. Early studies reported observation of spontaneous flagella-like rhythmic beating of kinocilia in vestibular HCs in frogs and eels (Flock et al., 1977; Rüsch and Thurm, 1990), as well as in zebrafish HCs in the early otic vesicle (Stooke-Vaughan et al., 2012; Wu et al., 2011) (see Movie 8 in reference 58).

We observed kinociliary motility in only a subset of the cells. There are two primary reasons why such motility has not been consistently observed in both current and previous investigations. First, HCs in vitro rapidly lose their physiological integrity when removed from their native electrochemical environment. This is especially critical for mammalian HCs, which depolarize quickly once this environment is disrupted. Second, the process of acute tissue dissection and handling may compromise the integrity of the kinociliary membrane, which—under native conditions—is straight and tethered to the overlying extracellular matrix. Such disruptions may impair kinocilia integrity and the metabolic conditions (such as depletion of ATP) essential for sustaining ciliary motility.

Although we observed kinocilium-driven bundle movement in adult mouse vestibular HCs, the magnitude of motility was substantially smaller than other motile cilia. Single-cell transcriptomic analysis showed the absence of 16 genes associated with the 96-nm axonemal repeat of human respiratory cilia, including *Pierce1* and *Pierce2*. These two MIPs-encoding genes have been shown to regulate motile cilia function and left–right asymmetry in mouse models (Gui et al., 2021; Sung et al., 2016). *Pierce1* knockout results in pronounced defects in ciliary motility and dynein arm docking, while *Pierce2* loss has a milder effect and largely preserves overall ciliary ultrastructure and beating (Gui et al., 2021; Sung et al., 2016). Although the contribution of these genes to kinociliary function remains uncertain, the absence of both may contribute to reduced microtubule stability or motor organization, especially when combined with additional losses in key motor components. However, MIPs are the most heterogeneous components across different types of cilia, such as sperm and airway cilia in different species (Leung et al., 2025; Tai et al., 2023). Thus, it is not clear if lack of expression of these two genes would lead to reduction of kinocilia motility in mouse vestibular HCs.

The lack of expression of two genes associated with IDAs (*Dnah12*, *Dnah3*) may lead to the loss of specific single-headed IDA components as suggested by our structural models (Fig. 8). Mutations of *DNAH3* and *DNAH12* are linked to male infertility and dynein dysfunction in humans (Meng et al., 2024; Yang et al., 2024). Mutations of DNAH12 cause male infertility by impairing DNAH1 and DNALI1 recruitment. However, it does not affect the tracheal tract and oviductal cilia organization (Yang et al., 2024). For the other two IDA-related genes (*Acta2, Cfap100*/*Ccdc37*) and one RS-related gene (*Morn3*), no ciliopathy has been linked to mutations of these three genes so far. We speculate that the lack of expression of DNAH3 and DNAH12 may be a key factor limiting kinocilia motility in mammalian vestibular HCs compared to respiratory cilia or kinocilia of nonmammalian species (e.g., bullfrog) HCs.

We detected the expression of few sperm-specific MIPs including *SAXO4*, *Tektin 3*, and *Tektin 4* in vestibular HCs. While the kinocilium shares a broader molecular profile with epithelial motile cilia, the presence of these distinct sperm-specific MIPs, which are absent from multi-ciliated epithelial tissues, suggests kinocilia may possess unique structural specializations adapted to their exceptional length (~60 – 70 μm in mouse crista HCs) and sensory function, highlighting unique identity of kinocilia.

The HC uses positive local feedback to amplify the inputs to their mechanosensitive hair bundles. This amplification is needed to overcome mechanical impedances or to fine tune stimulus. The remarkable sensitivity of the auditory system in mammals is attributed to the fast somatic motility of OHCs in the cochlea (Brownell et al., 1985; Dallos et al., 2008; Kachar et al., 1986; Liberman et al., 2002; Zheng et al., 2000). In other receptor organs, HCs may effect amplification by the Ca^2+^-dependent activity of myosin or transduction channels in the stereocilia (Fettiplace, 2017; Hudspeth, 1997). Transduction-mediated active hair bundle motion of turtle and frog HCs was reported before (Benser et al., 1996; Crawford and Fettiplace, 1985; Denk and Webb, 1992; Howard and Hudspeth, 1987; Martin et al., 2003). Our live imaging shows the kinocilia movement driving motion of the hair bundle (Fig. 7E, Videos S1 and S2). Therefore, the kinocilium may function as an active, force-generating hair bundle component to enhance mechanosensitivity of vestibular HCs. It is ideally positioned to modulate mechanical stimuli transmitted to the mechanosensitive stereocilia (Roberts et al., 1988). As the kinocilium is only connected to the top part of the tallest stereocilium by lateral links, the force generated by kinocilia motility is expected to be more effectively exerted to the tip-links to dynamically modulate tip-link tension and prime transduction channels. By contrast, this mode of active modulation has been supplanted by the somatic motility of OHCs in the mammalian cochlea (Brownell et al., 1985; Dallos et al., 2008; Kachar et al., 1986; Liberman et al., 2002; Zheng et al., 2000).

In summary, this study demonstrates that the kinocilium of vestibular HCs is a unique hybrid cilium, exhibiting near-complete molecular features of both primary and motile cilia. While it shares structural and molecular similarities with motile cilia and sperm flagella, it also possesses distinct architectural and functional characteristics. Future investigations employing kinocilium-specific proteomics, cryo-ET, and single-particle analysis will be critical for fully characterizing kinocilium molecular composition and organization. Although kinocilia motility was observed in bullfrog and mouse vestibular HCs in the present study, the mechanisms by which this motility influences mechanosensitivity of vestibular HCs remain to be elucidated.

## MATERIALS AND METHODS

Male and female CBA/J mice were purchased from the Jackson Laboratory (Stock #:000656) and reared in Animal Care Facility of Creighton University and NIDCD. American bullfrogs (*Rana catesbiana*) were purchased from Carolina Biological Supply Co. The animal usage and care were approved by the Institutional Animal Care and Use Committees of Creighton University (Protocol #1046.3) and NIDCD (NIDCD ACUC Protocol #1215).

### 1. Cell dissociation, cDNA libraries preparation and RNA-Sequencing

Male and female CBA/J mice aged 10 weeks were used for scRNA-seq. The cochlear and vestibular end organs (utricle, saccule, and crista) were dissected from the inner ear and placed in Petri dishes containing cold L-15 medium (Gibco; #11320033). After the cochlear and vestibular sensory epithelium and neurons were dissected out, they were separately transferred into two individual 1.5 ml tubes for enzymatic digestion (Collagenase IV from Sigma, concentration: 1mg/ml collagenase) in L-15 medium. After 10 minutes of incubation at room temperature, the enzymatic solution was removed and replaced with 400 μl L-15 media containing 10% fetal bovine serum. The tissues in two tubes were mechanically triturated by 200 μL Eppendorf pipette tips. After that, the suspension containing cochlear and vestibular cells was then passed through 40 μm strainers for filtration and pelleted at 300 g for 5 mins. After removing extra media, cells were then reconstituted in the 50 ul L-15 with 10% fetal bovine serum media and used for cDNA library preparation. Seven mice were used for each biological replicate. Six biological replicates for cochlear sensory epithelium and four biological replicates for vestibular sensory epithelia were prepared for scRNA-seq.

The emulsion droplets were constructed using a 10x Genomics Controller device following the manufacturer’s instruction manual. cDNA libraries were constructed using the 10x Genomics Chromium Single Cell 3’ Reagent Kits V3.1. High Sensitivity DNA Kits (Agilent Technologies) were used to perform quality control for each library in an Agilent 2100 Bioanalyzer. cDNA libraries were sequenced in an Illumina NextSeq 6000 sequencer aiming for 240 billion 150 bp long paired-end reads.

### 2. Single-cell RNA-seq data process and analysis

Raw transcriptomic datasets of adult cochlear and vestibular HCs from scRNA-seq have been deposited to GEO (GSE283534; reviewers access token: chqnuoeqzraxjyf). The FASTQ files were mapped to mm10 reference genome to generate the single-cell expression matrices following the CellRanger count pipeline (version 6.1.2). The Cellranger output data was then processed with the Seurat package (version 4.3.0) in R (version 4.1.3).

Genes expressed in at least ten cells were included in the analysis. Cells with numbers of expressed genes <200 or >3,000 and cells with numbers of unique molecular identifiers (UMI) > 15,000 were filtered out. Cells with >20% mitochondrial genes were also excluded from the analysis.

The gene expression data from individual samples were converted into a natural logarithm and normalized under the same condition. Data from six cochlear and four vestibular replicates were integrated separately based on the anchors identified from the top 2,000 highly variable genes of individual normalized expression matrices. The Shared Nearest Neighbor graph method can calculate the neighborhood overlap (Jaccard index) between every cell and its nearest neighbors, which was used for the cluster determination at a resolution of 0.6 on PCA-reduced expression data for the top 30 principal components.

Clustering results for cochlear and vestibular datasets were visualized separately using t-distributed stochastic neighbor embedding (t-SNE). Cluster annotations were primarily produced using SingleR and then corrected based on the differentially expressed genes (DEGs) for each cluster and the well-known cellular markers for cochlear and vestibular cells.

Entrez Gene, HGNC, OMIM, and Ensembl database were used for verification, reference, and analyses. Primary Cilium Proteome Database (https://esbl.nhlbi.nih.gov/Databases/CiliumProteome/), CiliaCarta (https://ngdc.cncb.ac.cn/databasecommons/database/id/6383), and Ciliogenics (https://ciliogenics.com/) were also used for reference.

GO analysis in Fig. S1 was performed using ShinyGO 0.82(Ge et al., 2020). The Venn diagrams in Fig. 5B and Fig. S3 were generated using Venny 2.1 (https://bioinfogp.cnb.csic.es/tools/venny/), except for the sperm Venn diagram in Fig. S3E, which was plotted using nVenn (https://bioinfogp.cnb.csic.es/tools/venny/). Fig. 5C was created with BioRender.com and further modified using Adobe Photoshop.

### 3. Immunocytochemistry

Inner ears were fixed overnight with 4% paraformaldehyde at 4^0^C. The cochlear and vestibular sensory epithelia were dissected out. After several washes with PBS, the tissue was blocked for 1 hour with 0.25% normal goat serum in PBS containing Triton-X-100 (0.01%) and goat serum (10%). Primary antibodies against DNM1 (AB3458, Abcam), SLC7A14 (HPA045929, Sigma), TJAP1 (NBP1-80902, Novus), FOXJ1 (14-9965-82, ThermoFisher), CCDC39 (HPA035364, Sigma), CCDC40 (PA5-54653, ThermoFisher), DNAH5 (31079-1-AP, ThermoFisher), DNAH6 (HPA036391, Sigma), TEKT1 (HPA044444, Millipore Sigma), CLUAP1 (PA5-83710, Thermo Fisher), IFT172 (28441-1-AP) and acetylated tubulin (T6793, Sigma) were incubated with the tissues for 12 hours at 4°C. After washes with PBS, secondary antibody (1:500) (Alexa fluor molecular probe 488 or 555; Invitrogen) was added and incubated for 2 hours at room temperature. Alexa Fluro^™^ 405 or 488 phalloidin (A30104 or A12379, Invitrogen) was used to label stereocilia bundles. Tissues were washed with PBS and mounted on glass microscopy slides with antifade solution (5 ml PBS, 5 ml glycerol, 0.1 g n-propylgallate). Images were captured using Nikon TI-2 Spinning Disk or Zeiss LSM 980 Inverted confocal microscope.

### 4. Single molecule fluorescence in situ hybridization

Single molecule fluorescence *in situ* hybridization was used to validate the expression of 15 genes in 10 μm-thin sections. Samples were prepared in formalin-fixed paraffin embedded tissue. Probes for 18 genes were purchased from ACD. These genes include *Adam11*, (580971), *Aqp11* (803751), *C1ql1* (465081), *Cdh23* (567261-C2), *Chrna10* (818521), *Cib2* (846681), *Cib3* (1105771), *Dnm3* (451841), *Ikzf2* (500001), *Kcnq4* (707481), *Otof* (485678), *Pcdh20* (467491), *Slc7a14* (544781), *Tmc1* (Cat No: 520911-C2) and *Tbx2* (448991-C2). Methods for the RNAscope^™^ 2.5 HD Assay from Advanced Cell Diagnostics were followed.

### 5. Electron microscopy

The mouse inner ears were fixed with 4% paraformaldehyde and 2.5% glutaraldehyde in 0.1 M sodium cacodylate buffer (pH 7.4) with 2 mM CaCl2. The bullfrog crista ampullaris was fixed with 3% paraformaldehyde, 2% glutaraldehyde, 2% tannic acid and 0.5% calcium chloride in 0.1 M sodium cacodylate buffer (pH 6.8) with 0.1 mM CaCl_2_ (Kachar et al., 1990). The tissues were then post-fixed for 1 hour with 1% OsO_4_ in 0.1 M sodium cacodylate buffer and washed. For SEM, cochleae and vestibular tissues were dehydrated via an ethanol series, critical point dried from CO_2_, and sputter-coated with platinum. The morphology of the HC stereocilia bundle was examined in a FEI Quanta 200 scanning electron microscope and photographed. For TEM, the bullfrog crista ampullaris was embedded in plastic (Epon 812 Epoxy Resin) after dehydration via an ethanol series. 70-nm thin sections were cut with a diamond knife and collected on 300-mesh grids. The thin section was post-stained with 3% uranyl acetate for 15 min and 1.5% lead citrate for 3 min. The preparations were examined in an electron microscope (JEOL 100CX) and photographed.

### 6. Measurements of ciliary motion

Bullfrogs were anesthetized with 20 μg/g of 3-aminobenzoic acid ethyl ester and decapitated. The saccular macula was carefully dissected out under cooled frog’s Ringer solution. The otoliths were gently removed with forceps in fresh Ringer solution(Jaeger et al., 1994). The saccular macula was placed under an Olympus upright microscope and kinocilia motility was visualized using DIC and 60x objective and captured with a camera.

The Eustachian tube was dissected out from CBA/J mice and sectioned along its longitudinal length. The preparation was bathed in L-15 medium (Invitrogen), containing 136 mM NaCl, 5.8 mM NaH_2_PO_4_, 5.4 mM KCl, 1.4 mM CaCl_2_, 0.9 mM MgCl_2_, 0.4 mM MgSO_4_, and 10 mM HEPES-NaOH (pH 7.4, 300 mmol/l) in an experimental chamber mounted on the stage of a Leica upright microscope. Crista ampullaris was also dissected from 10-week-old CBA/J mice and bathed in L-15 medium. The tissue was attached to the bottom of the chamber by the weight of two thin platinum rods (0.5 mm in diameter). The tissue was mounted with the cilia or hair bundles facing upward toward the water-immersion objective. The cilia and hair bundles were imaged using a 63x water immersion objective (Leica) and magnified by an additional 10x relay lens. Ciliary motion was measured and calibrated by a photodiode-based measurement system mounted on the Leica upright microscope (Jia and He, 2005). The magnified image of the cilia was projected onto a photodiode through a rectangular slit. Cilia/bundle motion modulated the light influx to the photodiode. The photocurrent response was calibrated to displacement units by moving the slit a fixed distance (0.5 μm) with the image of the cell in front of the photodiode. After amplification, the photocurrent signal was low pass-filtered by an antialiasing filter before being digitized by a 16-bit A/D board (Digidata 1550A; Molecular Devices). The motile responses were low-pass filtered at 250 Hz and digitized at 1 kHz. Ciliary motion was acquired in a two-second window for each trial and 20 trials were captured for each cell in one recording. The power spectrum of the response was averaged and analyzed in the frequency domain using Clampfit software (version 10, Molecular Devices). The experiments were performed at room temperature (22±2°C).

### 7. Predicted model of the 96-nm modular repeat in adult vestibular kinocilia

The 96-nm repeat structures of the human respiratory axoneme (PDB: 8J07) and bovine sperm flagellum (PDB: 9FQR) were used as structural references to model the vestibular kinocilia axoneme. Candidate genes enriched in vestibular HCs, identified through transcriptomic profiling were annotated and mapped to their respective axonemal compartments based on known or predicted protein localizations within these reference structures. The 8J07 model lacked atomic modeling for the full-length radial spoke 3 (RS3)(Zhao et al., 2025), despite its presence in the corresponding cryo-EM density map (EMD-35888), due to the uncharacterized proteome of RS3 in the human respiratory system. To address this, RS3 components from the sperm axoneme structure (PDB: 9FQR) excluding sperm-specific proteins were extracted, fitted into the EMD-35888 density using ChimeraX’s “fit-to-map” tool, and overlaid onto the 8J07 model to complete the RS3 architecture. All structural visualization and model integration were performed using UCSF ChimeraX v1.6. (Pettersen et al., 2021). In the models, DNAH5 and DNAH9 (present in our HC data) occupy the ODA region in human respiratory cilia, while DNAH8 and DNAH17 occupy the same region in sperm (but are not expressed in our dataset). Since they correspond structurally, we did not mark them as missing in the sperm-based kinocilia model.

## Supplementary Material

Supplement 1 **Table S1** (Transcriptomes of four HC types)
**Fig. S1:** GO analysis of enriched genes in cellular localization and biological processes primarily related to cilia organization, assembly, maintenance, intracellular transport, and microtubule dynamics particularly associated with motile cilia
**Fig. S2:** Primary cilia-related genes in four HC types
**Fig. S3**: Comparison of HC transcriptomes with multi-tissue proteomics datasets
**Fig. S4**: ATAC-seq data indicating enhanced accessibility of motile-cilia machinery-associated gene loci in adult vestibular HCs
**Fig. S5**: Microtubule inner proteins (MIPs) and microtubule-associated proteins (MAPs) absent from mouse HC scRNA-seq data

Supplement 2 **Table S1** (Transcriptomes of four HC types)

Supplement 3 **Videos S1 and S2**: Bullfrog kinocilia motility

Supplement 4
