# Supplementary material for: The Dual Molecular Identity of Vestibular Kinocilia: Bridging Structural and Functional Traits of Primary and Motile Cilia
Source: bioRxiv. 2025 Jun 23:2025.04.16.647417. Preprint. [Version 3] doi: 10.1101/2025.04.16.647417 (PMC12190475; doi:10.1101/2025.04.16.647417)
Supplement: Supplement 1 — Table S1 (Transcriptomes of four HC types) Fig. S1: GO analysis of enriched genes in cellular localization and biological processes primarily related to cilia organization, assembly, maintenance, intracellular transport, and microtubule dynamics particularly associated with motile cilia Fig. S2: Primary cilia-related genes in four HC types Fig. S3: Comparison of HC transcriptomes with multi-tissue proteomics datasets Fig. S4: ATAC-seq data indicating enhanced accessibility of motile-cilia machinery-associated gene loci in adult vestibular HCs Fig. S5: Microtubule inner proteins (MIPs) and microtubule-associated proteins (MAPs) absent from mouse HC scRNA-seq data [file media-1.pdf]

Supplemental Figures for Manuscript by Xu et al:

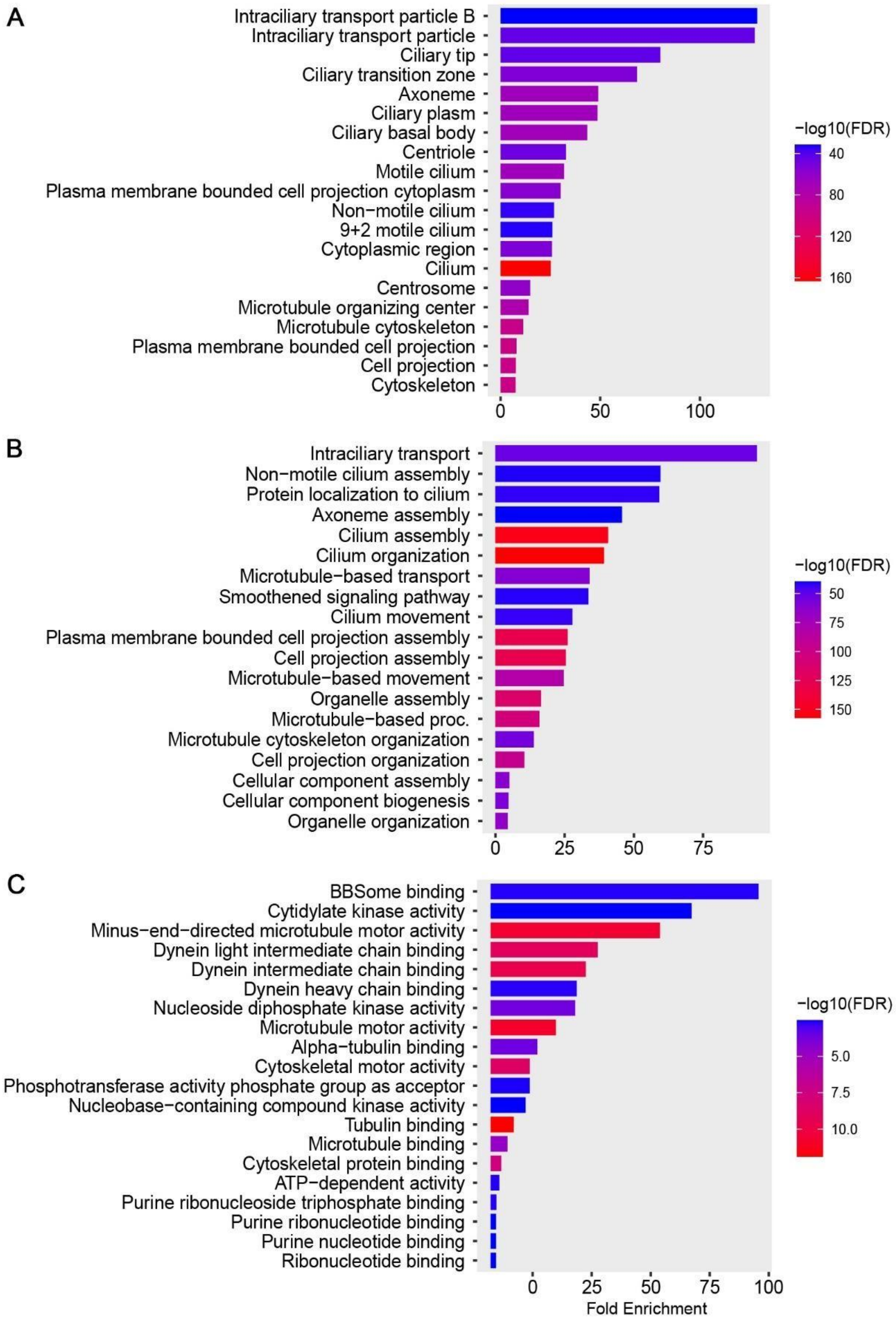

Fig. S1: Gene Ontology (GO) analysis of shared genes in panel 5B across multiple ciliary databases and the current study. (A) Cellular localization, (B) Molecular function, and (C) Biological processes. The analysis reveals enrichment in terms related to cilia organization, assembly,

maintenance, intracellular transport, and microtubule dynamics, particularly those associated with motile cilia.

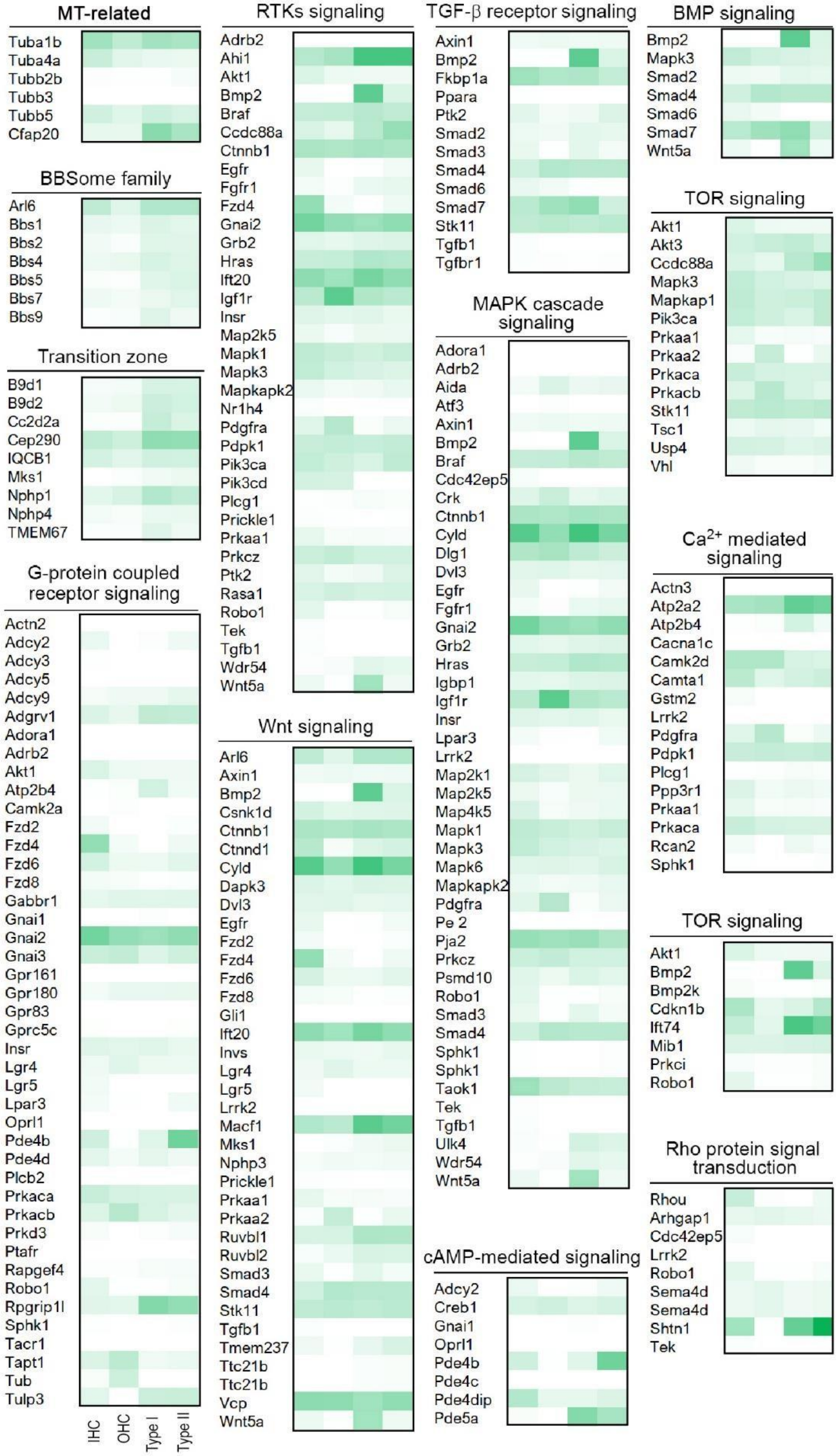

Fig. S2: Primary cilia-related genes in four HC subtypes. Overlap of shared genes involved in cilia maintenance, including microtubule-associated proteins, BBSome family members, and

components of the transition zone and primary cilia signaling pathways associated with primary cilia and their basal body.

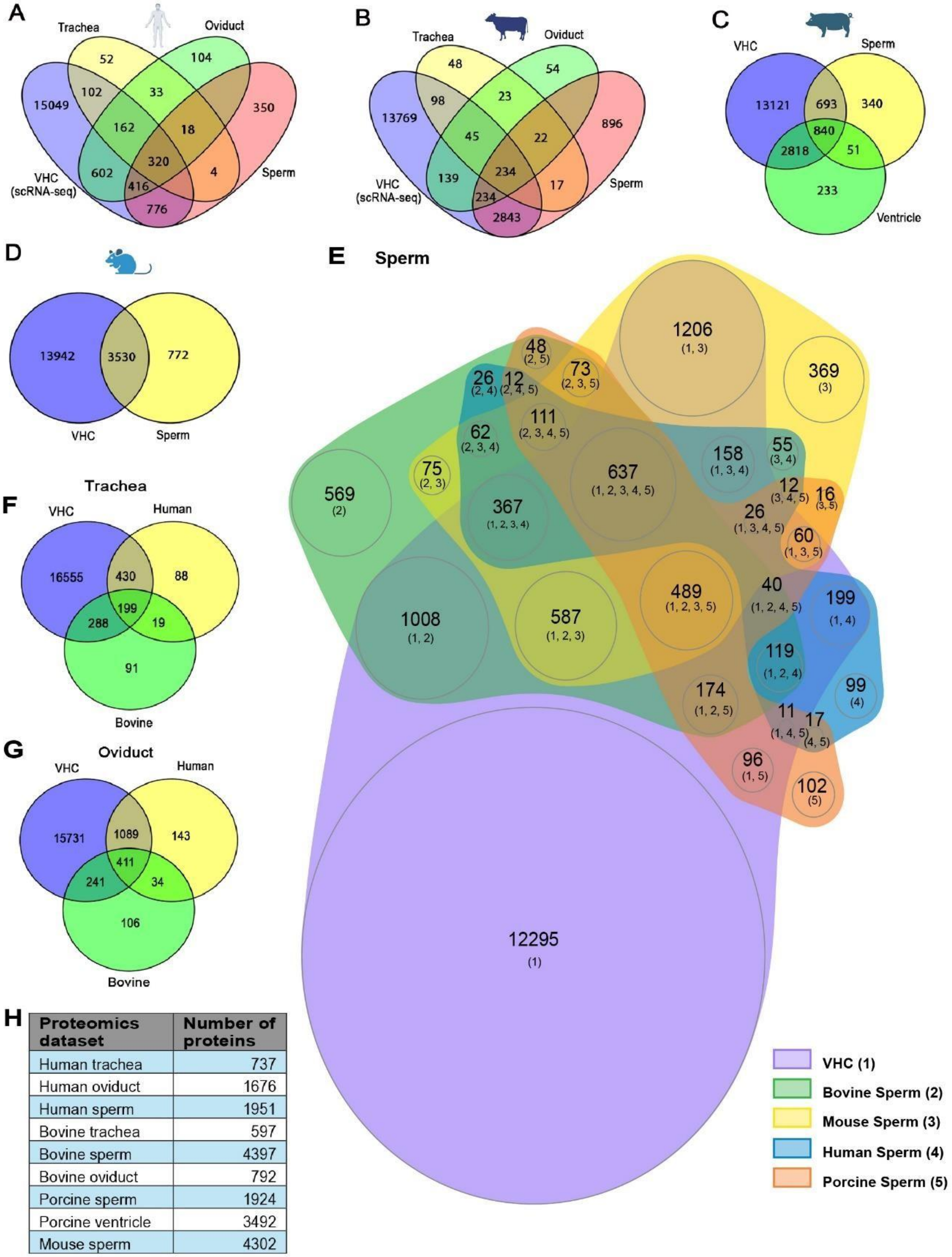

Proteomics dataset	Number of proteins
Human trachea	737
Human oviduct	1676
Human sperm	1951
Bovine trachea	597
Bovine sperm	4397
Bovine oviduct	792
Porcine sperm	1924
Porcine ventricle	3492
Mouse sperm	4302

Fig. S3: Comparison of vestibular HC scRNA-seq data with proteomics datasets from multiple tissues and species containing motile cilia. (A–D) Cross-species comparisons of motile cilia proteomes from different tissues, each compared with VHC scRNA-seq data: (A) Human trachea, oviduct, and sperm. (B) Bovine trachea, oviduct, and sperm. (C) Porcine sperm and ventricle. (D) Mouse sperm. (E–G) Tissue-specific comparisons across species, each compared with VHC scRNA-seq data: (E) Sperm proteomes from bovine, mouse, human, and porcine. (F)

Tracheal proteomes from human and bovine. (G) Oviduct proteomes from human and bovine. (H) Number of proteins identified in each dataset included in the comparisons.

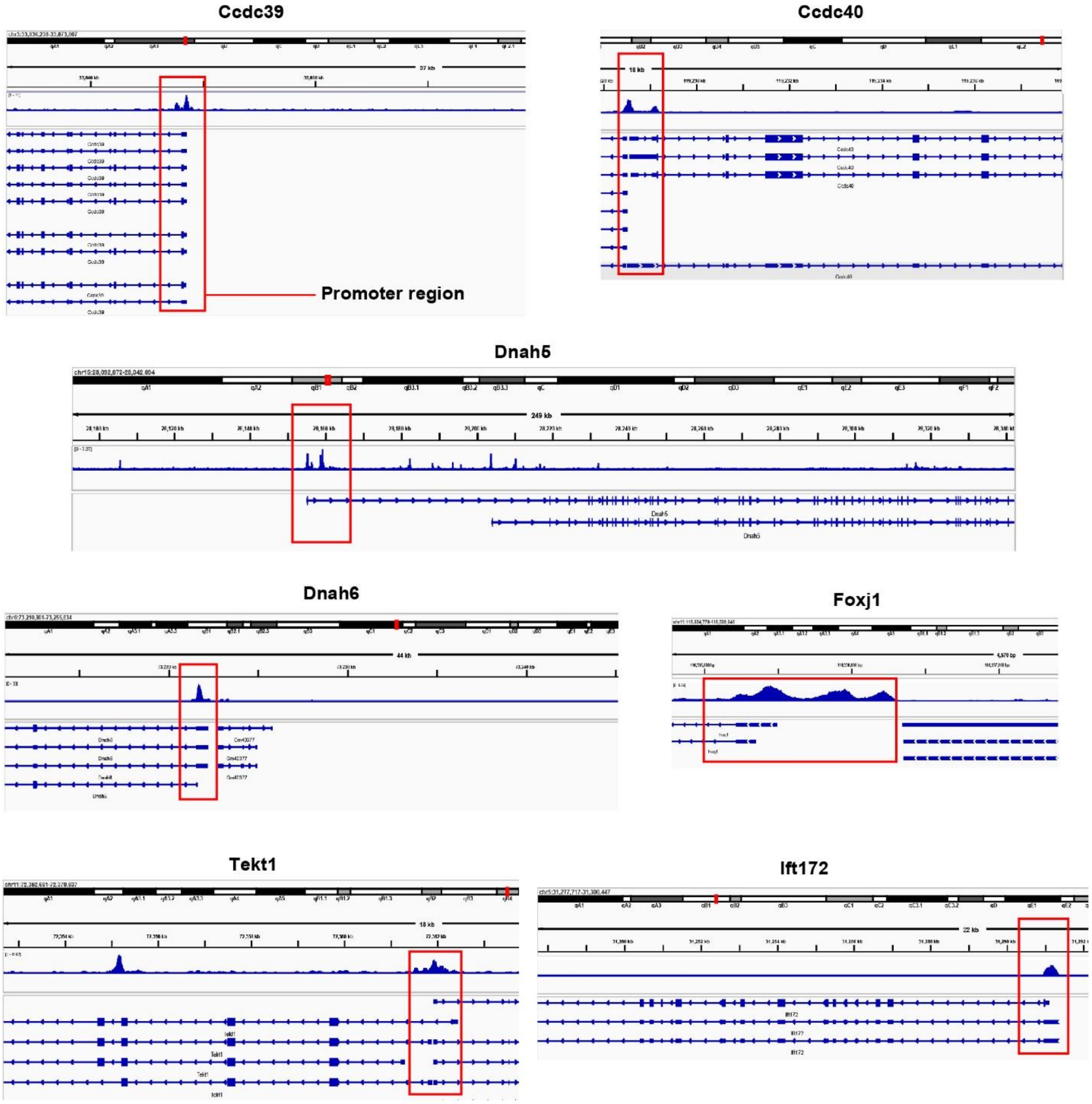

Fig. S4: Enhanced accessibility of motile cilia-associated gene loci in adult vestibular HCs based on published ATAC-seq data

Axonemal complex	Missing genes	Corresponding PDB model	Effect on motility/Cilia-related disease
MIPs and MAPs	CFAP68	8J07/9FQR	unknown
	CFAP90	8J07/9FQR	unknown
	CFAP95	8J07/9FQR	unknown
	CFAP107	8J07/9FQR	unknown
	CFAP141	8J07/9FQR	unknown
	CFAP276	8J07/9FQR	unknown
	FAM166B	8J07/9FQR	unknown
	FAM183A	8J07/9FQR	unknown
	FAM166C	8J07/9FQR	unknown
	Pierce1	8J07/9FQR	LR asymmetry<br>Defect on ciliary motility and laterality
	Pierce2	8J07/9FQR	LR asymmetry<br>Defect on ciliary motility and laterality
	SPMIP1	9FQR	unknown
	CIMIP1	9FQR	unknown
	SPMAP1	9FQR	unknown
	CFAP 144	9FQR	unknown
	CFAP 263	9FQR	unknown
	CIMAP1B	9FQR	unknown
	CIMAP1C	9FQR	unknown
	CIMIP3	9FQR	unknown
	CCDC105	9FQR	unknown
	DUSP21	9FQR	unknown
	EFCAB3	9FQR	unknown
	ODF3	9FQR	unknown
	SIMIP4	9FQR	unknown
	SPMIP7	9FQR	unknown
	SPMIP5	9FQR	unknown
	SPMIP6	9FQR	unknown
	SAXO3	9FQR	unknown
	TEKTIP1	9FQR	unknown
	Tektin-5	9FQR	unknown
	SPMIP8	9FQR	unknown
	TEX33	9FQR	unknown
	TEX49	9FQR	unknown
	TSSk6	9FQR	unknown
	TEX37	9FQR	unknown
	THEGL	9FQR	unknown
	CFAP 96	9FQR	unknown
	CFAP 299	9FQR	unknown

Fig. S5: Microtubule inner proteins (MIPs) and microtubule-associated proteins (MAPs) absent from mouse HC scRNA-seq data. These genes structurally resolved in the 96-nm axonemal repeats of human respiratory and/or bovine sperm flagella (PDB: 8J07, 9FQR). While most of these proteins are not known to directly regulate motility, a few such as Pierce1 and Pierce2 are linked to defects in ciliary motility and left–right (LR) asymmetry.
